# Non-canonical Hedgehog Signaling Pathway in Cancer: Activation of GLI Transcription Factors Beyond Smoothened

**DOI:** 10.3389/fgene.2019.00556

**Published:** 2019-06-12

**Authors:** Silvia Pietrobono, Sinforosa Gagliardi, Barbara Stecca

**Affiliations:** Tumor Cell Biology Unit – Core Research Laboratory, Institute for Cancer Research, Prevention and Clinical Network (ISPRO), Florence, Italy

**Keywords:** Hedgehog, GLI, cancer, non-canonical, oncogene, tumor suppressor, targeted therapy

## Abstract

The Hedgehog-GLI (HH-GLI) pathway is a highly conserved signaling that plays a critical role in controlling cell specification, cell–cell interaction and tissue patterning during embryonic development. Canonical activation of HH-GLI signaling occurs through binding of HH ligands to the twelve-pass transmembrane receptor Patched 1 (PTCH1), which derepresses the seven-pass transmembrane G protein-coupled receptor Smoothened (SMO). Thus, active SMO initiates a complex intracellular cascade that leads to the activation of the three GLI transcription factors, the final effectors of the HH-GLI pathway. Aberrant activation of this signaling has been implicated in a wide variety of tumors, such as those of the brain, skin, breast, gastrointestinal, lung, pancreas, prostate and ovary. In several of these cases, activation of HH-GLI signaling is mediated by overproduction of HH ligands (e.g., prostate cancer), loss-of-function mutations in *PTCH1* or gain-of-function mutations in *SMO*, which occur in the majority of basal cell carcinoma (BCC), SHH-subtype medulloblastoma and rhabdomyosarcoma. Besides the classical canonical ligand-PTCH1-SMO route, mounting evidence points toward additional, non-canonical ways of GLI activation in cancer. By non-canonical we refer to all those mechanisms of activation of the GLI transcription factors occurring independently of SMO. Often, in a given cancer type canonical and non-canonical activation of HH-GLI signaling co-exist, and in some cancer types, more than one mechanism of non-canonical activation may occur. Tumors harboring non-canonical HH-GLI signaling are less sensitive to SMO inhibition, posing a threat for therapeutic efficacy of these antagonists. Here we will review the most recent findings on the involvement of alternative signaling pathways in inducing GLI activity in cancer and stem cells. We will also discuss the rationale of targeting these oncogenic pathways in combination with HH-GLI inhibitors as a promising anti-cancer therapies.

## Introduction

Hedgehog-GLI (HH-GLI) signaling is a conserved pathway that plays critical roles during embryonic development, cellular proliferation, differentiation and stem cell maintenance (Ingham and McMahon, 2001). The mammalian apparatus of the HH-GLI pathway consists of three secreted HH ligands, Sonic Hedgehog (SHH), Indian Hedgehog (IHH) and Desert Hedgehog (DHH), the 12-pass transmembrane receptor Patched 1 (PTCH1), the 7-pass transmembrane G protein-coupled receptor (GPCR) Smoothened (SMO), as the obligatory signal transducer across the plasma membrane, and the three GLI transcription factors (GLI1, GLI2 and GLI3), as the executors of the transcriptional response of HH signaling (Varjosalo and Taipale, 2008).

**FIGURE 1:**
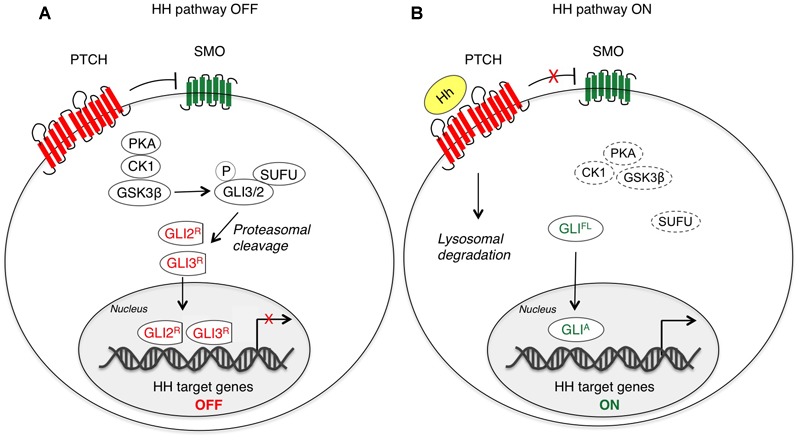
Canonical activation of HH-GLI signaling. In absence of the HH ligand **(A)**, PTCH inhibits SMO, and therefore GLI2 and GLI3 are phosphorylated by PKA, CK1 and GSK3β, which create binding sites for the E3 ubiquitin ligase β-TrCP. GLI3 and GLI2 undergo partial proteasome degradation, leading to the formation of repressor forms (GLI3/2^R^), that translocate into the nucleus where they inhibit the transcription of HH target genes. Upon HH ligand binding **(B)**, the repression of SMO by PTCH is relieved, allowing accumulation and activation of SMO. Thus, SMO promotes a signaling cascade that ultimately leads to translocation of full length (FL) activated forms of GLI (GLI^A^) into the nucleus, where they induce transcription of HH target genes. CK1, caseine kinase 1; GLI2/3^R^, GLI2/3 repressors; GLI^A^, GLI activators; GLI^FL^, GLI full length; GSK3β, glycogen synthase kinase 3β; Hh, Hedgehog; PKA, protein kinase A; PTCH, Patched; SMO, Smoothened; SUFU, Suppressor of Fused.

Hedgehog-mediated transduction is initiated by binding of HH ligands to PTCH. In absence of ligand, PTCH localizes at the base of the primary cilium, an organelle that bulges from the surface of vertebrate cells acting as a signaling center specialized for HH signal transduction (Goetz and Anderson, 2010). Thus, the accumulation of SMO to the cilium is prevented and its activity is repressed (Denef et al., 2000; Rohatgi et al., 2007). In this condition, protein kinase A (PKA), casein kinase 1 (CK1) and glycogen synthase kinase 3β (GSK3β) phosphorylate GLI2 and GLI3, which are recognized by the F-box protein β-transducing repeat-containing protein (β-TrCP) and sequestered in the cytoplasm through the physical interaction with Suppressor of Fused (SUFU) that prevents their nucleo-cytoplasmic shuttling (Kogerman et al., 1999; Wang and Li, 2006; Pan and Wang, 2007; Niewiadomski et al., 2014). In the cytoplasm, they are subjected to proteolytic cleavage to generate C-terminally truncated forms (GLI^R^) that repress transcription of HH target genes (Pan et al., 2006). In contrast to GLI2 and GLI3, GLI1 is not cleaved into a repressor form, but it is mainly regulated at transcriptional level. GLI1 protein can be also controlled by the ubiquitin-proteasome system (UPS) that recognizes two degradation signals (degrons) within the GLI1, one on the C-terminus (degron D_C_) and the other on N-terminus (degron D_N_), preventing inappropriate signaling activity (Huntzicker et al., 2006) (Figure 1A).

Activation of the HH-GLI pathway is triggered by binding of HH ligand that displaces PTCH1 from the primary cilium, thus inducing its internalization and lysosomal degradation (Rohatgi et al., 2007). This allows SMO to be phosphorylated and activated through its association with CK1α and GPCR kinase 2 (GRK2) (Chen et al., 2011) and to translocate to the cilium likely through its association with β-arrestin (Corbit et al., 2005; Kovacs et al., 2008; Chen et al., 2011). Active SMO translates the extracellular HH stimulus that prevents GLI2 and GLI3 processing and promotes their dissociation from SUFU, leading to translocation of full-length and active GLI (GLI^A^) into the nucleus, where they activate transcription of HH target genes, including factors involved in cell proliferation, survival, self-renewal and invasiveness. Among targets there is also GLI1 itself (Katoh and Katoh, 2009; Gupta et al., 2010), which represents a reliable marker for HH pathway activation and requires functional GLI2 and GLI3 for its transcriptional activation (Dai et al., 1999; Ikram et al., 2004) (Figure 1B).

The HH-GLI signaling pathway is required during embryonic development and tissue homeostasis. After birth HH-GLI signaling is turned off and it remains active only in specific progenitor/stem cells, where it is involved in tissue repair and regeneration. However, its abnormal reactivation during childhood or the adult life can lead to cancer. Persistent activation of HH-GLI signaling has been reported in several cancers, including solid tumors and hematological malignancies, where it has been associated with tumor development, progression and recurrence after chemotherapy via the regulation of residual cancer initiating cells (Hanna and Shevde, 2016). Deregulated HH-GLI signaling can be the result of (i) ligand-independent activation due to inactivating mutations in *PTCH1* or *SUFU*, activating mutations in *SMO*, or *GLI* gene amplifications (i.e., in basal cell carcinoma (BCC), SHH-subtype medulloblastoma, rhabdomyosarcoma); (ii) autocrine/juxtacrine ligand-dependent activation, in which tumor cells increase HH ligand expression and respond to the same HH stimulation in a cell-autonomous manner (i.e., glioblastoma, melanoma, lung, breast, stomach and prostate cancers); (iii) paracrine ligand-dependent activation, where HH ligands secreted by tumor cells turn on HH signaling in the surrounding stroma, which, in turn, stimulates growth and survival of the tumor and vice versa (i.e., pancreatic and colorectal cancers) (reviewed in Barakat et al., 2010; Teglund and Toftgård, 2010; Amakye et al., 2013; Cochrane et al., 2015).

However, cumulative evidence indicates that regulation of GLI expression and activity may occur also in response to other signaling pathways besides PTCH-SMO, reducing therapeutic efficacy of SMO antagonists. In this review we will focus on additional modes of GLI activation in cancer and cancer stem cells (CSCs) that occur independently of SMO. The existence of these non-canonical mechanisms appears relevant to allow the development of novel therapeutic approaches to eradicate tumors dependent on HH-GLI signaling.

## The GLI Transcription Factors

**FIGURE 2:**
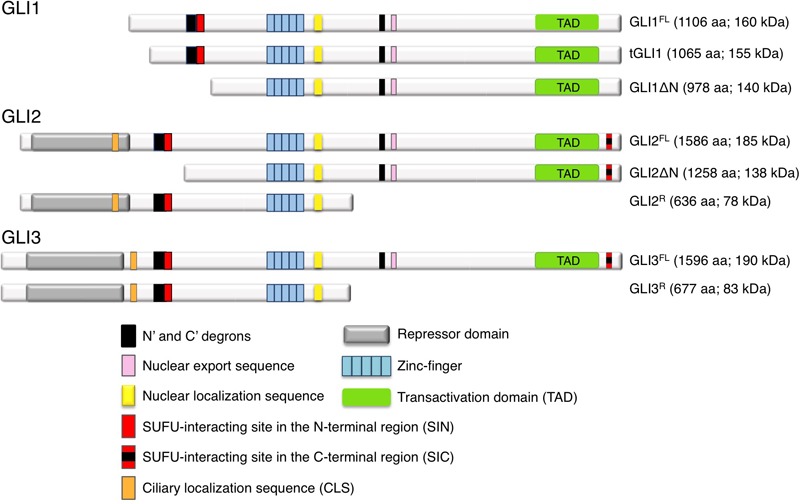
Schematic representation of human GLI1, GLI2, and GLI3 isoforms. See text for details.

GLI proteins are members of the Gli-Kruppel family of zinc-finger (ZNF) containing transcription factors (TFs), with five C_2_H_2_-Kruppel type ZNF motifs constituting the specific DNA binding domain. ZNF4 and ZNF5 bind specifically to a 9 base pair DNA consensus sequence (9-mer) 5′-GACCACCCA-3′ within the GLI-target gene promoters (Kinzler and Vogelstein, 1990), whereas ZNF1-3 contribute to stabilize the DNA binding domain by interacting with the phosphate backbone (Pavletich and Pabo, 1993). A nuclear export sequence (NES) and a canonical bipartite nuclear localization signal (NLS), the latter adjacent to the fifth ZNF domain, ensure the nucleo-cytoplasmic shuttling of GLI (Bauer et al., 2015) (Figure 2). Although the three GLI TFs bind the 9-mer with similar affinity, different GLI can preferentially activate target genes in a context-dependent manner. Indeed, only the two cytosine-pairs flanking the central adenine within the consensus site are critical for GLI binding, whereas the other positions can tolerate a certain degree of flexibility (Winklmayr et al., 2010). Further, epigenetic changes in the regulatory regions of GLI target genes, the presence of specific GLI co-factors or the cooperation with other transcription factors can alter the DNA binding affinity of GLI to their targets and affect the transcriptional output (Regl et al., 2004; Asaoka et al., 2010; Peterson et al., 2012).

All GLI proteins possess a SUFU-interacting site located on their N-terminus (SIN) (Han Y. et al., 2015), which is responsible for SUFU-mediated cytoplasmic retention of GLI1. GLI2 and GLI3 contain an additional SUFU-interacting site on their C-terminus (named SIC) (Han Y. et al., 2015), that appears to be required for the inhibition of GLI transcriptional activity in the nucleus. All GLI proteins also possess a C-terminal transactivation domain (TAD), but GLI2 and GLI3 have also a N-terminal repressor domain that allows them to function as both transcriptional activators and repressors depending on cellular context, although GLI3 has been reported as a strong repressor in most settings (Tsanev et al., 2009). Thus GLI1 acts mainly as transcriptional activator (Carpenter and Lo, 2012), whereas full-length GLI2 is generally a weak activator, since the fully activated form requires the complete removal of its N-terminus (Roessler et al., 2005; Speek et al., 2006; Grachtchouk et al., 2011; Pantazi et al., 2014). A second conserved NLS containing a ciliary localization signal (CLS) has been recently identified within the N-terminal region of GLI2 and GLI3. This site has been suggested to be involved in GLI^A^ formation without altering their proteolytic processing into GLI^R^ (Han et al., 2017). Abnormal activation of GLI^A^ and GLI1 represents a critical parameter for both tumor initiation and progression (Tojo et al., 2003; Carpenter and Lo, 2012; Iwasaki et al., 2013; Sadam et al., 2016).

The human *GLI1* gene was first identified by Vogelstein and colleagues as a putative oncogene amplified in glioblastoma (Kinzler et al., 1987), and its overexpression has been reported in several tumors, including those of breast, colon, lung, ovarian, pancreas and prostate, in BCC, medulloblastoma, glioblastoma, meningioma and melanoma, where it regulates genes involved in proliferation, angiogenesis, epithelial-to-mesenchymal transition (EMT), invasiveness, CSC renewal and drug resistance (Kasper et al., 2006; Teglund and Toftgård, 2010; Aberger et al., 2012; Palle et al., 2015; Mastrangelo and Milani, 2018). In the last decade, two isoforms of GLI1, namely GLI1ΔN and tGLI1, have been described (Figure 2). The N-terminal deletion variant (GLI1ΔN) is a product of alternative splicing between exon 1 and exon 4. This event generates a protein with a 128-amino acid deletion on its N-terminus. This deletion prevents both SUFU binding and the degradation signal of GLI1 degron D_N_, while preserving the ZNF domains, NLS and NES, and the transactivation domain. Despite loss of SUFU-binding domain, GLI1ΔN is a labile activator and acts on target genes similarly to full-length GLI1 (GLI1^FL^). Further, it does not show a preferential expression in cancer tissues (Shimokawa et al., 2008). Conversely, the truncated GLI1 (tGLI1) originates from a splicing of exon 3 and part of exon 4 of the *GLI1* gene, resulting in the deletion of 41 amino acids (Lo et al., 2009). This isoform retains all the functional domains present in GLI1^FL^ and translocates into the nucleus to activate gene transcription. However, contrarily to GLI1^FL^ and GLI1ΔN, tGLI1 shows a tumor-specific expression, and appears to regulate an additional set of target genes involved in EMT, invasion and metastasis (Lo et al., 2009; Cao et al., 2012). Similar to GLI1^FL^, both these alternative spliced proteins (GLI1ΔN and tGLI1) respond to HH ligand stimulation, but whether and how they interact with the other components of the pathway has not yet been determined.

## Non-Canonical Activation of GLI Transcription Factors in Cancer and Stem Cells

**FIGURE 3:**
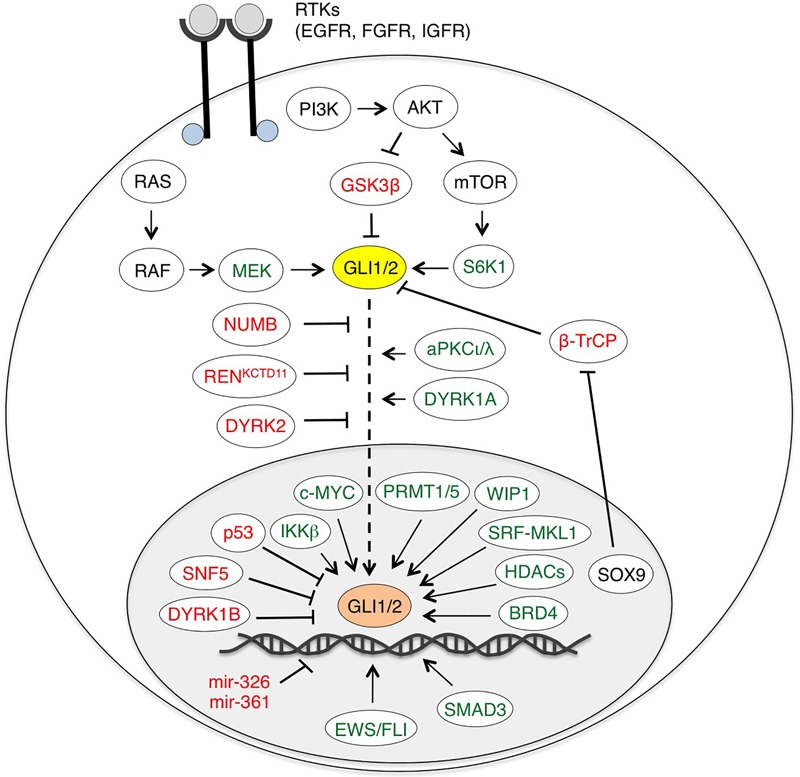
Non-canonical activation of HH-GLI signaling by oncogenic pathways. Schematic diagram of GLI1/2 and their positive (green) and negative regulators (red). EWS/FLI, SMAD3, miR-326 and miR-361 are only direct transcriptional regulators of GLI1/2. BRD4, HDACs and SRF-MKL1 regulate both GLI1/2 expression and transcriptional activity. See text for details. Abbreviations: AKT, protein kinase B; aPKCι/λ, atypical protein kinase Cι/λ; BRD4, bromodomain-containing protein 4; β-TrCP, β-transducin repeat-containing protein; DYRK1/2, dual specificity tyrosine-phosphorylation-regulated kinase 1/2; EWS/FLI, Ewing Sarcoma/Friend Leukemia Integration 1; GSK3β, glycogen synthase kinase 3β; HDAC, histone deacetylase; IKKβ, inhibitor of nuclear factor kappa-B kinase subunit β; MEK, MAPK (Mitogen-activated protein kinase)/ERK (extracellular signal-regulated kinase) kinase; PI3K, phosphoinositide 3-kinase; PRMT1/5, protein arginine methyltransferases 1/5; SRF-MKL1, serum response factor-megakaryoblastic leukemia 1; S6K1, ribosomal protein S6 kinase 1; WIP1, wild-type p53-induced phosphatase 1.

In addition to the canonical activation of GLI by the HH-PTCH-SMO route, which is typical of normal cells and ciliated tumors, growing evidence points to a SMO-independent stimulation of GLI activity in cancer. These non-canonical mechanisms are involved both in transcriptional activation of GLI genes and in post-translational modifications of GLI proteins (summarized in Table 1), representing a heterogeneous mosaic of alterations that contribute to the development of several types of cancer with elevated GLI activity (Brechbiel et al., 2014) (Figure 3).

**Table 1:** Mechanisms of non-canonical activation of Hedgehog-GLI signaling.

Upstream Regulator	Mechanism of action	Cancer/Cell type	References
**RAS-RAF-MEK-ERK**			
MEK1/2-ERK1/2	Increases expression of Gli target genes; Gli1 required for KRAS-driven transformation	KRAS-driven PDAC mouse model	Nolan-Stevaux et al., 2009
	Increases GLI1/2 transcriptional activity	NIH3T3	Riobò et al., 2006a
		Melanoma	Stecca et al., 2007
		PDAC	Ji et al., 2007
		Gastric cancer	Seto et al., 2009
		Colon cancer	Mazumdar et al., 2011
		LAC	Po et al., 2017
	Increases GLI1 nuclear localization	Melanoma	Stecca et al., 2007
	Induces GLI1 protein stability	PDAC	Ji et al., 2007
	Induces GLI2 protein stability	BCC	Kasper et al., 2006
MEK1/2-RSK2	Promotes GLI2 nuclear localization and stabilization	Multiple myeloma	Liu et al., 2014
**MAPKKK/MEKK**			
MEKK1	Inhibits GLI1 transcriptional activity	MB	Antonucci et al., 2019
MEKK2/3	Inhibits GLI1 transcriptional activity and protein stability through SUFU	MB	Lu et al., 2018
**PI3K/AKT/mTOR**			
AKT	Increases Gli2 transcriptional activity	NIH3T3	Riobò et al., 2006b
	Increases GLI1 transcriptional activity and nuclear translocation	Melanoma	Stecca et al., 2007
	Enhances GLI1 protein stability	PDAC, ovarian cancer	Singh et al., 2017
	Prevents GLI degradation (GSK3β-dep.)	ALCL	Singh et al., 2009
mTOR/S6K1	Enhances GLI1 activation preventing SUFU association	EAC	Wang et al., 2012
p70S6K2	Prevents GLI1 degradation (GSK3β-dep.)	NSCLC	Mizuarai et al., 2009
		EAC	Kebenko et al., 2015
**TGFβ**	Increases GLI2 transcription (SMAD3-dep.)	PDAC, BC	Dennler et al., 2007, 2009
	Increases GLI2 expression	Colon CSC	Tang et al., 2018
	Stimulates GLI1 transcriptional activity (PCAF-dep.)	PDAC	Nye et al., 2014
**PKC signaling**			
PKCα	Reduces GLI1 transcriptional activity	HEK-293T	Neill et al., 2003
	Increases GLI1 transcriptional activity	Hep3B, NIH3T3	Cai et al., 2009
PKCδ	Increases GLI1 transcriptional activity	HEK-293T	Neill et al., 2003
	Reduces GLI1 transcriptional activity	Hep3B, NIH3T3	Cai et al., 2009
aPKCι/λ	Enhances DNA binding and GLI1 transcriptional activity	BCC	Atwood et al., 2013
**DYRK family**			
DYRK1A	Promotes GLI1 nuclear translocation	NIH3T3, HEK-293T	Mao et al., 2002; Shimokawa et al., 2008; Ehe et al., 2017
	Induces GLI1 degradation, mediated by F-actin and MKL1	Lung carcinoma, rhabdomyosarcoma	Schneider et al., 2015
DYRK1B	Enhances GLI1 transcriptional activity	PDAC, MB	Gruber et al., 2016
DYRK2	Induces GLI2 protein degradation	NIH3T3	Varjosalo et al., 2008
**Oncogenic drivers**			
EWS/FLI1	Induces GLI1 transcription	Ewing sarcoma	Zwerner et al., 2008; Beauchamp et al., 2009
SOX9	Prevents βTrCP-mediated GLI1 degradation	Pancreatic CSC	Deng et al., 2015
FOXC1	Enhances GLI2 transcriptional activity	Basal-like BC	Han B.C. et al., 2015
c-MYC	Enhances GLI1 transcription	Burkitt lymphoma	Yoon et al., 2013
IKKβ	Promotes GLI1 stability	DLBCL	Agarwal et al., 2016
SRF-MKL1	Induces GLI transcription and enhances DNA binding	BCC	Whitson et al., 2018
WIP1	Enhances GLI1 transcriptional activity, nuclear localization and protein stability	Melanoma	Pandolfi et al., 2013
**Tumor suppressors**			
p53	Inhibits GLI1 transcriptional activity, nuclear translocation and protein stability	Glioblastoma	Stecca and Ruiz i Altaba, 2009
	Promotes proteasome-dependent degradation of GLI1 (PCAF-dep.)	MB	Mazzà et al., 2013
	Interferes with DNA binding ability of GLI1 (TAF9-dep.)	Rhabdomyosarcoma, Osteosarcoma	Yoon et al., 2015
NUMB	Induces GLI1 ubiquitination and proteasome degradation (ITCH-dep.)	MB	Di Marcotullio et al., 2006, 2011
SNF5	Interferes with promoter occupancy of GLI1	Rhabdoid Tumors	Jagani et al., 2010
**miRNAs**			
miR-324-5p	Represses GLI1 expression	CGCPs	Ferretti et al., 2008
miR-361	Represses GLI1 expression	Prostate cancer	Chen et al., 2017
	Represses GLI1 and GLI3 expression	Retinoblastoma and CSC	Zhao and Cui, 2019
miR-326	Represses GLI2 expression	Ptch+/- MB CSC	Miele et al., 2017
**BET proteins**			
BRD4	Increases GLI1/2 transcription	BCC	Tang et al., 2014
		MB	Long et al., 2014
BET	Upregulates Gli1 in murine CAFs	PDAC	Yamamoto et al., 2016
BET	Promotes GLI occupancy on target promoters	PDAC	Huang et al., 2016
**HDAC**			
HDAC	Stimulates GLI1 nuclear localization and transcriptional activity	Multiple Myeloma	Geng et al., 2018
HDAC class I	Increases DNA binding ability of GLI1 (HDAC1)	MB MB, murine BCC	Canettieri et al., 2010 Gruber et al., 2018
HDAC class II	Transcriptional control of GLI2 (HDAC6)	MB	Dhanyamraju et al., 2015
**HAT**			
p300	Prevents GLI2 recruitment to chromatin	HEK-293T, NIH3T3	Coni et al., 2013
PCAF	Acts as GLI1 transcriptional cofactor	Glioblastoma, MB	Malatesta et al., 2013
	Promotes GLI1 ubiquitination and proteolysis	MB	Mazzà et al., 2013
**PRMTs**			
PRMT1	Enhances DNA binding ability of GLI1	PDAC	Wang et al., 2016
PRMT5	Enhances GLI1 protein stabilization and nuclear translocation	C3H10T1/2, HEK-293T, SCLC	Abe et al., 2019
	Inhibits GLI1 expression through Menin1	Neuroendocrine tumors	Gurung et al., 2013

*ALCL, anaplastic large cell lymphoma; β-TrCP, β-transducing repeat-containing protein; BC, breast cancer; BCC, basal cell carcinoma; BET, bromo- and extra-terminal domain; CAF, cancer-associated fibroblats; CSC, cancer stem cells; DLBCL, diffuse large B-cell lymphoma; DYRK, dual-specificity tyrosine phosphorylation-regulated kinase; EAC, esophageal adenocarcinoma; CGCPs, cerebellar granule cell precursors; GSK3β, glycogen synthase kinase 3β; HAT, histone acetyltransferase; HDAC, histone deacetylase; LAC, lung adenocarcinoma; MEF, mouse embryonic fibroblasts; NSCLC, non-small cell lung cancer; PCAF, p300/CREB-binding protein-associated factor; PDAC, pancreatic ductal adenocarcinoma; PKC, protein kinase C; PRMT, protein arginine methyltransferases; SCLC, small cell lung cancer; SUFU, Suppressor of Fused.*

### RAS-RAF-MEK-ERK Signaling

The RAS-RAF-MEK-ERK pathway plays pivotal roles in several cellular processes, such as cell proliferation and growth. Its constitutive activation is caused by mutations or altered stimulation of pathway components (mainly RAS, RAF, MEK) and leads to the hyperactivation of the mitogen-activated protein kinase (MAPK) extracellular signal-regulated kinase 1 and 2 (ERK1/2), which promote tumor cell growth (Samatar and Poulikakos, 2014). Several reports have described a cross-talk between the HH-GLI and RAS-RAF-MEK-ERK signaling pathways (e.g., Rovida and Stecca, 2015).

The first hint of a positive regulation of HH pathway by the RAS-RAF-MEK-ERK signaling came from a study showing that constitutively active MEK1 increases GLI1 activity, which is totally abrogated by inhibition of MEK1/2. Authors identified a region containing the first 130 amino acids of GLI1 responsible for sensing the status of ERK1/2 signaling. Indeed, its deletion yields to an active GLI1 protein showing decreased response to MEK1 signaling (Riobò et al., 2006a). A subsequent computational analysis identified a putative MAPK consensus site in the N-terminal region of GLI (Whisenant et al., 2010). However, evidence that ERK1/2 directly phosphorylates GLI1 is missing, suggesting the potential involvement of an additional kinase downstream of ERK1/2.

A number of reports have shown that the RAS-RAF-MEK-ERK signaling can modulate the HH pathway in several types of cancer. In human melanoma cells, oncogenic NRAS (NRAS^Q61K^) and HRAS (HRAS^V 12G^) are able to enhance transcriptional activity and nuclear localization of GLI1. The cytoplasmic retention of GLI1 by SUFU is prevented by both oncogenes. Since inhibition of MEK1/2 abolishes the effect of oncogenic RAS on GLI1, it is likely that the MEK1/2-ERK1/2 module acts as the main effector of RAS (Stecca et al., 2007).

The HH-GLI pathway is important for the initiation and progression of pancreatic adenocarcinoma (PDAC), which is also characterized by high incidence of activating *KRAS* mutations (70–90% of cases) (Morton et al., 2007). KRAS can activate HH-GLI signaling in PDAC. Indeed, in the immortalized human PDAC cell line HPDE-c7, oncogenic KRas^V 12^ increases GLI1 transcriptional activity and protein levels. KRAS-mediated GLI1 activation is insensitive to cyclopamine inhibition, suggesting that is independent of SMO. GLI1 protein stability and HH pathway activation are decreased by pharmacological inhibition of MEK1/2 with UO126 (Ji et al., 2007). Interaction between KRAS and HH signaling has also been described in mouse models of PDAC. Oncogenic KRAS (KRAS^G12D^) cooperates with a dominant active form of Gli2 that lacks the N-terminal repressive domain to initiate PDAC tumorigenesis *in vivo* (Pasca di Magliano et al., 2006). Another mouse model of KRAS-induced PDAC shows that SMO-independent activation of GLI1 is required for survival of mouse and human PDAC cells and KRAS-mediated transformation *in vitro* (Nolan-Stevaux et al., 2009).

In gastric cancer, KRAS positively modulates tumor proliferation increasing GLI1 transcriptional activity and expression of HH target genes. EGF-induced stimulation of GLI is insensitive to SMO inhibition and is blocked by MEK1 inhibition (Seto et al., 2009). Similarly, the RAS-RAF pathway induces GLI1 and GLI2 transcriptional activity and increases mRNA and protein levels in a non-canonical manner in colon cancer cells. Pharmacological and genetic inhibition of GLI function is more effective in reducing tumor proliferation and inducing apoptosis than the inhibition of the canonical pathway at SMO level, suggesting that GLI activity is crucial for RAS/MEK-induced colon cancer proliferation (Mazumdar et al., 2011).

In lung adenocarcinoma (LAC) cells and in their CSC compartment, GLI1 is active despite SMO is often epigenetically silenced. Consistently, genetic silencing or pharmacological inhibition of GLI1, but not of SMO, reduces LAC tumor growth and stemness *in vitro* and *in vivo* (Po et al., 2017). Mechanistically, GLI1 is phosphorylated and activated in a SMO-independent manner by MAPK-ERK1/2 signaling, that can be induced by KRAS or through stimulation of Neuropilin 2 receptor by its ligand VEGFA, secreted by cancer cells in an autocrine loop or by stromal cells in a paracrine manner. The cross-talk between epithelial and stromal cells appears to be mediated by the expression and secretion of SHH by LAC epithelial cells, which activates canonical HH-GLI signaling in stroma, increasing transcription of VEGFA and other GLI1 target genes (Po et al., 2017).

In multiple myelomas, the MEK1/RSK (ribosomal S6 kinase) cascade sustains non-canonical HH pathway activation through the stabilization of GLI2. RSK2 promotes GLI2 nuclear localization and impairs its degradation through inhibitory phosphorylation of GSK3β on Ser9 (Liu et al., 2014). In these cells, abnormal HH-GLI signaling correlates with the high activity of MEK/RSK signaling compared to normal B cells. Combined treatment with the RSK inhibitor SL0101 and the GLI inhibitor GANT58 reduces myeloma cell survival and HH-GLI pathway activation with a synergistic effect (Liu et al., 2014).

Various upstream growth factor receptor tyrosine kinases (RTK) can have an impact on GLI activity independent of SMO, through the activation of the RAS-RAF-MEK1/2-ERK1/2 signaling. In human normal keratinocytes, co-expression of EGF and GLI factors enhances the activation of the HH-GLI pathway and promotes oncogenic transformation activating the MEK1/2-ERK1/2 pathway (Schnidar et al., 2009). Integration of these two signaling pathways converges in the regulation of HH/EGFR response genes, such as SOX2, SOX9, JUN, CXCR4, and FGF19, which are cooperatively induced in response to HH and EGFR signaling activation in BCC and in tumor-initiating pancreatic cells (Eberl et al., 2012). EGFR signaling modulates GLI target genes expression by regulating GLI1 and GLI2 interaction with their transcriptional cofactors at the promoter of their target genes (Kasper et al., 2006). MEK1/2-ERK1/2 activity upon EGFR stimulation is crucial to integrate HH/EGFR signaling. Indeed, ERK1/2 activates the JUN/AP-1 genes, which cooperate with GLI proteins to activate downstream target genes responsible for BCC cell transformation. Moreover, activation of ERK1/2 by EGF leads to the stabilization of GLI factors, especially GLI2, preventing its proteasomal degradation (Kasper et al., 2006). Genetic and pharmacological inhibition of EGFR and MEK signaling confirms this mechanism also in medulloblastoma (Götschel et al., 2013) and in prostate cancer (Zhu et al., 2013).

Surprisingly, recent studies reported that the apical members of the MAPK cascade, the mitogen activated kinases MEKK1 and MEKK2/3, exert a negative action on GLI1. Indeed, MEKK1 overexpression strongly decreases transcriptional activity of GLI1, causing the inhibition of HH-GLI signaling and reduction of cell growth and viability of medulloblastoma cells. MEKK1 directly phosphorylates multiple residues in the GLI1 C-terminal region, promoting the association with the 14-3-3 cytoplasmic protein and reducing GLI1 transcriptional activity (Antonucci et al., 2019). The reason of the opposite regulation of GLI1 by MEKK1 and its downstream target MEK1/2 is not clear, and further studies are required to address this issue, which is crucial to devise an effective targeted therapy. To date, only one study reports that the MEKK1/MEK1/ERK1 cascade positively regulates GLI1 in human lung fibroblasts (Cheng et al., 2016). In medulloblastoma cells MEKK2/3 mediates the negative modulation of FGF on HH pathway, by interacting with GLI1 and suppressing HH-dependent medulloblastoma cell growth. MEKK2 and MEKK3 bind and phosphorylate GLI1 on multiple Serine/Threonine residues (Ser201, Ser204, Ser243, Ser968, Thr1074, Ser1078), reducing GLI1 protein stability, DNA-binding and promoting its association with SUFU (Lu et al., 2018).

### PI3K-AKT-mTOR Signaling

The PI3K-AKT-mTOR signaling contributes to a variety of cellular processes, such as nutrient uptake, proliferation and survival in both physiological and pathological conditions, including cancer (Yu and Cui, 2016). The first evidence of the involvement of PI3K signaling in non-canonical GLI activation came in 2006, when Riobò et al. (2006b) demonstrated that Akt prevents proteasomal degradation of Gli2 by antagonizing the inhibitory effect of PKA, thus facilitating Gli2 activation and nuclear translocation. Activation of PI3K-AKT signaling has been found to enhance GLI1 protein stability in pancreatic and ovarian cancer (Singh et al., 2017) and in anaplastic large cell lymphoma (ALCL), where AKT1 counteracts the inhibitory effect of GSK3β on GLI1 (Singh et al., 2009). In addition, AKT stimulates GLI1 transcriptional activity and nuclear translocation in human melanoma, prostate cancer and glioma cells, contributing to disease progression (Stecca et al., 2007). Consistently, genetic silencing of AKT or pharmacological inhibition of PI3K-AKT signaling downregulates GLI1 in human esophageal adenocarcinoma (EAC) cells (Kebenko et al., 2015). Furthermore, the PI3K-AKT-NFκB axis has been shown to mediate the activation of GLI2 in stromal cells by the cytokine CCL5. In turn, GLI2 induces transcriptional activation of IL-6, which binds the IL-6R on malignant B cells, leading to immunoglobulin hyperproduction (Elsawa et al., 2011).

PI3K signaling has been also reported to increase GLI1 activity via members of the ribosomal S6 kinase family (S6K/p70-S6K), which are the downstream effectors of the PI3K-AKT-mTOR axis. For instance, in EAC cells, mTOR activation by TNFα is responsible for S6K1 phosphorylation and activation. Activated S6K1 directly phosphorylates GLI1 at Serine residue at position 84, thus preventing its association with SUFU (Wang et al., 2012). Another member of the S6K family, p70-S6K2, has been shown to inhibit GSK3β through an inhibitory phosphorylation at residue Ser9, thus preventing GSK3β-mediated GLI1 degradation (Mizuarai et al., 2009). However, the role of PI3K-AKT-mTOR in regulating GLI1 in cancer appears to be context dependent. Indeed, studies in neuroblastoma showed that S6K1 fails to modulate GLI1 activity (Diao et al., 2014), and, surprisingly, GLI1 acts as tumor suppressor in this context and its tumor-suppressive functions are inhibited by AKT2 (Paul et al., 2013).

Smoothened-independent and PI3K-dependent non-canonical GLI activation has been related to increased *in vitro* proliferation and clonogenicity, and *in vivo* tumor growth in squamous cell lung tumors (Kasiri et al., 2017), renal cell carcinoma (Zhou et al., 2016) and colon cancer (Cai et al., 2015). The finding that heterozygous ablation of PTEN in a mouse model of medulloblastoma carrying a SmoA1 transgene promotes medulloblastoma formation (Castellino et al., 2010), strongly supports the importance of PI3K signaling as therapeutic target. In line with these data, administration of the dual PI3K/mTOR inhibitor NPV-BEZ-235 has been shown to suppress GLI1-dependent cancer cell proliferation in androgen-independent prostate cancer cells (Yang et al., 2017). Moreover, combination of NPV-BEZ-235 with the SMO inhibitor NVP-LDE-225 demonstrated enhanced efficacy than single agents in the inhibition of self-renewal and tumorigenicity of pancreatic CSCs (Sharma et al., 2015).

Upregulation of PI3K signaling has been linked to acquired resistance to SMO inhibitors for the re-expression of GLI1 in medulloblastoma (Buonamici et al., 2010), and proposed as a potential resistance mechanism for other tumors such as esophageal cancer (Wang et al., 2012), implying that a combinatorial approach targeting both canonical (with SMO inhibitors) and non-canonical GLI activation (via PI3K inhibitors) could delay tumor resistance (Buonamici et al., 2010; Dijkgraaf et al., 2011).

### TGF-β Signaling

Transforming growth factor β (TGF-β) signaling exerts important functions during embryonic development and homeostasis of adult tissues. During carcinogenesis, TGF-β signaling can act both as tumor suppressor or oncogene depending on tumor type and stage (David and Massagué, 2018). An increasing number of reports shows that TGF-β signaling can interact with the HH-GLI pathway downstream of SMO. Dennler et al. (2007) were the first to demonstrate that TGF-β pathway regulates GLI transcription factors in a SMO-independent manner in human normal fibroblasts and keratinocytes, as well as in PDAC and breast cancer cells. The induction of *GLI2* by TGF-β occurs through a SMAD3-dependent mechanism and mediates subsequent activation of *GLI1.* Interestingly, TGF-β signaling is required for GLI expression and proliferation of cyclopamine-resistant pancreatic carcinoma cells. In these cells, treatment with an antagonist of the TGF-β receptor (TβRI) reduces *GLI2* mRNA levels and cell proliferation (Dennler et al., 2007). A further study showed that GLI2 is a direct transcriptional target of TGF-β, that induces a rapid increase of GLI2 expression independently of PTCH/SMO, through the cooperation of SMAD3 and β-catenin on *GLI2* promoter (Dennler et al., 2009). In addition, TGF-β is able to stimulate GLI1 activity in cancer cells and to induce a subset of TGF-β-inducible target genes, including BCL2, IL7 and Cyclin D1 (Nye et al., 2014). GLI1 interacts with SMAD proteins to modulate TGF-β-induced gene expression dependent on p300/CREB-binding protein-associated factor (PCAF) (Nye et al., 2014).

Several mouse models have shown that TGF-β signaling is also critical for Hh-mediated carcinogenesis. For instance, in a model of pancreatic cancer, SMO-independent Gli1 activation promotes transformation and requires both TGF-β and KRAS signaling (Nolan-Stevaux et al., 2009). Similarly, TGF-β signaling appears to be required in a mouse model of Smo-driven BCC, where inhibition of TGF-β by TβRI antagonist SD208 significantly reduces tumor burden and increases infiltration of lymphocytes. The relevance of this finding is mirrored in human BCCs, which often harbor activation of both HH and TGF-β signaling, as shown by increased phosphorylation of SMAD2 and SMAD3 (Fan et al., 2010).

Non-canonical activation of GLI by TGF-β plays a driving role in EMT and invasion of different tumors. In melanoma, high GLI2 levels are associated with a metastatic phenotype with appearance of mesenchymal features both *in vitro* and *in vivo*. Consistently, GLI2 silencing reduces invasion of extracellular matrix *in vitro* and bone metastasis in mice (Alexaki et al., 2010). In a metastasis model of breast cancer cells MDA-MB-231, SMO-independent induction of GLI2 is required for TGF-β to stimulate the expression of parathyroid hormone-related protein (PTHrP), an important osteolytic factor in bone metastasis (Johnson et al., 2011). In these cells, *SMO* is not detectable and the SMO inhibitor cyclopamine fails to repress GLI2. Furthermore, blocking HH signaling in metastatic breast cancer cells with the repressor form of GLI2 reduces endogenous and TGF-β-stimulated PTHrP expression and bone metastasis in mice (Johnson et al., 2011), supporting the role of GLI2 downstream of TGF-β in driving metastasis.

The interaction between TGF-β and HH-GLI signaling is also associated to cancer cell stemness and chemoresistance. Indeed, recurrent ovarian tumors are enriched in CSCs that express high levels of GLI2 and components of TGF-β pathway, such as the co-receptor endoglin (CD105). Interestingly, GLI2 inhibition sensitizes cells to cisplatin treatment and reduces tumor proliferation, and both CD105 and GLI2 are proposed as promising targets to overcome resistance (Steg et al., 2012). In colorectal cancer, hypoxia-inducible factor (HIF-1α) and cancer-associated fibroblasts (CAFs)-secreted TGF-β2 converge to induce strong expression of GLI2 in CSCs, independently of HH signaling (Tang et al., 2018). HIF-1α/TGF-β2/GLI2 expression promotes stemness and chemoresistance in colorectal cancers and is associated with patient relapse following chemotherapy. Interestingly from a therapeutic point of view, combined inhibition of both TGF-β with SD208 and GLI2 with GANT61 restores chemoresistance, reducing both self-renewal and survival of CSCs (Tang et al., 2018).

### PKC Signaling

The protein kinase C (PKC) is a serine/threonine kinase consisting of three members: calcium-dependent conventional PKC (cPKC; isoforms α, βI, βII and γ), calcium-independent novel PKC (nPKC; isoforms δ, 𝜀, η and 𝜃), and calcium-independent atypical PKC (aPKC; isoforms ζ and ι/λ). The role of PKCα and PKCδ in controlling GLI activity is controversial. Constitutive activation of PKCα reduces the transcriptional activity of GLI1, whereas that of PKCδ enhances GLI1 transcriptional activity in HEK-293T cells independent of MAPK signaling (Neill et al., 2003). In contrast, another report showed that in NIH3T3 cells and in human hepatoma Hep3B cells PKCα increases GLI1 transcriptional activity through MEK1/2-ERK1/2 pathway activation (Cai et al., 2009). Conversely, wild-type and constitutive active PKCδ functions as negative regulator of HH pathway, reducing GLI1 mRNA level and transcriptional activity. PKCδ co-immunoprecipitates with GLI1, but the interaction does not involve its kinase activity (Cai et al., 2009). Riobò et al. (2006a) found that PKCδ mediates the phorbol esters activation of Hh pathway, increasing Gli1 transcriptional activity through MEK1/2 activation.

On the other hand, aPKCι/λ activates GLI1 downstream of SMO, through phosphorylation of two residues (Ser243 and Thr304) in the zinc finger DNA binding domain of GLI1, leading to increased DNA binding and transcriptional activity (Atwood et al., 2013). Furthermore, GLI1 promotes the transcription of the gene encoding for aPKCι, contributing to form a positive GLI-aPKCι regulatory loop. Interestingly, activated aPKCι/λ is upregulated in BCC resistant to the SMO inhibitor vismodegib and targeting aPKCι/λ suppresses the HH pathway and growth of resistant BCC cell lines (Atwood et al., 2013). In addition, Justilien et al. (2014) demonstrated that PKCι phosphorylates SOX2, a transcriptional regulator of stemness, promoting the autonomous transcription of HH ligand. The functional interaction between PKCι and SOX2 coordinately drives growth and maintenance of lung squamous cell carcinoma stem-like cells.

### DYRK1 and 2

Dual-specificity tyrosine phosphorylation-regulated kinases (DYRKs) are serine, threonine and tyrosine kinases containing a DYRK-homology box. Among the five DYRK members, DYRK1A and DYRK1B play a dual role in GLI regulation, whereas DYRK2 is mainly an inhibitor. Overexpression studies indicated that DYRK1A can act as a positive regulator of GLI1, promoting its nuclear translocation (Mao et al., 2002; Shimokawa et al., 2008) through direct phosphorylation of GLI1 nuclear localization clusters in the N-terminus (Ser102/104/130/132) and at Ser408 (Schneider et al., 2015; Ehe et al., 2017). In contrast, DYRK1A is also able to induce GLI1 degradation by repressing the transcriptional co-activator megakaryoblastic leukemia 1 (MKL1), independent of upstream PTCH/SMO signaling. The final decision about which signaling prevails appears to be cell-type specific or depends on DYRK1A and GLI1 expression levels (Schneider et al., 2015).

DYRK1B can act as activator or repressor. It inhibits GLI2 function and promotes the formation of the GLI3 repressor form (Lauth et al., 2010). Conversely, DYRK1B can increase GLI1 activity, and its inhibition has been shown to repress GLI1 expression in both SMO-inhibitor sensitive and resistant cells (Gruber et al., 2016). Another study showed that DYRK1B can promote AKT-mediated GLI1 stability (Singh et al., 2017). The other DYRK member, DYRK2, phosphorylates Gli2 on two conserved Serine residues (at position Ser385 and Ser1011), promoting its proteasome-dependent degradation (Varjosalo et al., 2008).

### AMPK

5′ Adenosine monophosphate (AMP)-activated protein kinase (AMPK) is a serine/threonine kinase that supervises cellular energy status in response to the nutrient supply and environmental conditions; it is activated by increased AMP/ATP ratio and controls different energetic processes (i.e., growth, metabolism, protein synthesis). Activated AMPK phosphorylates GLI1 at Serine/Threonine residues (Ser102, Ser408 and Thr1074), decreasing both transcriptional activity and protein stability (Li et al., 2015). However, another report indicates that only phosphorylation at Ser408 is critical for GLI1 degradation and appears to reduce HH-driven cell growth in human medulloblastoma (Di Magno et al., 2016). Furthermore, AMPK has been shown to increase GLI1 cytoplasmic localization and to promote its interaction with the E3 ubiquitin ligase β-TrCP, leading to GLI1 degradation by the proteasome (Zhang et al., 2017).

AMPK can also activate the HH-GLI pathway, acting downstream of SMO to stimulate metabolic reprogramming toward glycolysis in adipocytes, and increases glucose uptake (Teperino et al., 2012). In addition, another report showed that AMPK mediates a non-canonical HH signaling that promotes polyamine metabolism, by activating an axis that leads to translation of ornithine decarboxylase (ODC) (D’Amico et al., 2015). In response to Hh activation, AMPK phosphorylates and activates the zinc finger protein CNBP (cellular nucleic acid-binding protein), which increases its association with Sufu, followed by CNBP stabilization, ODC translation, and polyamine biosynthesis. Interestingly, targeting this axis efficiently blocks Hh-dependent proliferation of medulloblastoma cells *in vitro* and *in vivo* (D’Amico et al., 2015). These findings suggest that AMPK can favor or inhibit tumorigenesis in a context-dependent manner. Therefore, further preclinical and clinical studies are required to warrant the use of AMPK modulators in anti-cancer therapy.

### Oncogenic Drivers

Activation of GLI transcription by oncogenes represents an additional mode of SMO-independent modulation. For instance, the Ewing Sarcoma/Friend Leukemia Integration 1 (EWS/FLI1) fusion oncogene, that characterizes Ewing Sarcoma Family Tumors (ESFT), has been shown to induce *GLI1* transcription via direct binding to *GLI1* promoter, with a consequent increase in the expression of activated GLI1 protein (Zwerner et al., 2008; Beauchamp et al., 2009). Indeed, ESFT show deregulated expression of GLI1 (Joo et al., 2009) and this appears critical for the ESFT phenotype, since genetic or pharmacological inhibition of GLI1 reduces the EWS/FLI1 transformation activity, impairs *in vitro* cell proliferation and colony formation, and abrogates *in vivo* tumor growth (Zwerner et al., 2008; Beauchamp et al., 2009).

In addition, the SRY-related high mobility group (HMG) box transcription factor SOX9 has been reported to regulate GLI1 by suppressing its association with β-TrCP and subsequent ubiquitination (Deng et al., 2015). Indeed, SOX9 binds the N-terminal F-box domain of β-TrCP through its transactivation domain and tethers β-TrCP into the nucleus, protecting nuclear GLI1 from degradation. This mechanism is critical for promoting SOX9-dependent CSC-like properties of pancreatic cancer cells (Deng et al., 2015).

Han and colleagues showed that the Forkhead box C1 (FOXC1) stimulates SMO-independent HH signaling in basal-like breast cancer (BLBC) by binding GLI2 (Han B.C. et al., 2015). This interaction occurs through the N-terminal domain of FOXC1 and is predicted to allosterically open the DNA binding domain of GLI2. This favors the transcriptional activity of GLI2, leading to increased CSC properties through the induction of a stem cell-like signature in BLBC cells. In support of this, increased FOXC1 levels have been detected in SMO inhibitor-resistant cells, whereas its depletion reverts the resistant phenotype.

Further, Yoon et al. (2013) showed that the oncogene c-MYC enhances the expression of GLI1 in Burkitt lymphoma cells, by direct interaction with the E-box (CANNTG) within the 5′ regulatory region of *GLI1*. Recently, the beta subunit of the IKK complex (IKKβ), which is induced in response to TNFα, has been reported to increase GLI1 protein levels and transcriptional activity in diffuse large B-cell lymphoma (Agarwal et al., 2016). The authors identified 8 IKKβ-dependent phosphorylation sites within the C-terminus of GLI1, which include a cluster of 6 Serine residues (S543-S548), a Serine residue at position 1071 and a Threonine residue at 1074. IKKβ-mediated phosphorylation of these sites prevents the association of GLI1 with the E3 ubiquitin ligase ITCH responsible for GLI1 degradation, thus favoring its stabilization (Agarwal et al., 2016).

Non-canonical GLI regulation by oncogenes has been also related to the development of resistance toward SMO inhibitors in cancer. By using a multidimensional genomic analysis of mouse and human drug-resistant BCCs, Whitson et al. (2018) found that the serum response factor (SRF) and its transcriptional co-factor MKL1 form a protein complex with GLI1 that enhances the transcription of HH-pathway target genes. Further, SRF-MKL1 also directly regulates GLI1 and GLI2 expression by binding to their 5′-untranslated regions (5′-UTR), thus inducing amplification of GLI1 transcriptional activity and drug-resistant BCC growth (Whitson et al., 2018).

The oncogenic wild-type p53-induced phosphatase 1 (WIP1) has been shown to cooperate with HH signaling to increase tumor formation in SHH-dependent medulloblastoma (Doucette et al., 2012). Similarly, mice overexpressing WIP1 under the control of the *Neurod2* promoter present increased proliferation in the external cellular layer of the cerebellum through activation of the endogenous Hh signaling (Wen et al., 2016). Interestingly, medulloblastoma incidence increases when ND2:WIP1 mice are crossed with SmoA1, whereas Wip1 knockout suppresses MB formation in two Hh-dependent mouse model of medulloblastoma (Wen et al., 2016). These studies demonstrated an interplay between Wip1 and Hh signaling in medulloblastoma development. Our group identified the mechanism of regulation of GLI by WIP1 in melanoma cells. We found that WIP1 enhances GLI1 nuclear localization, protein stability and transcriptional activity, without affecting GLI2 nor GLI3 (Pandolfi et al., 2013). WIP1 phosphatase activity appears to modulate GLI1 transcriptional activity, suggesting a direct dephosphorylation of GLI1 by WIP1, although evidence of a direct dephosphorylation is still lacking (Pandolfi et al., 2013).

Non-canonical activation of the HH pathway can also be mediated by the interaction of the androgen receptor (AR) with the GLI transcription factors. A recent report showed that transcriptionally active AR increases GLI transcriptional activity in prostate cancer cells. AR binds to the GLI3 protein processing domain at the C-terminus, impairing GLI3 ubiquitination and degradation (Li et al., 2018). AR prevents the formation of GLI3 repressor and stabilizes its activator form, promoting HH signaling. A truncated polypeptide of 270 amino acids derived from GLI2 and containing the AR binding site, is able to compete with GLI3 for binding to AR, inhibiting HH-GLI signaling in prostate cancer cells (Li et al., 2018).

### Tumor Suppressors and miRNAs: Negative Regulators of the GLI

In addition to the various oncogenic signals that positively influence GLI activity, increasing evidence suggests that tumor suppressive signals play also a crucial role in the control of GLI function and activity.

The tumor suppressor p53 controls cellular homeostasis and protects cells from tumorigenic events by inducing apoptosis, cellular senescence and cell cycle arrest in response to different cellular stress signals, such as DNA damage, hypoxia or oncogenic activation. Importantly, over half of human cancers are characterized by loss-of-function mutations in the *TP53* gene (Bieging et al., 2014). Several studies pointed to a negative regulation of GLI by p53. Stecca and Ruiz i Altaba (2009) reported that p53 restrains GLI1-driven neural stem cell self-renewal, and glioblastoma cell growth and proliferation. p53 represses GLI1 activity, nuclear localization and levels. p53 has been also found to affect the phosphorylation status of the GLI1ΔN isoform, acting either directly or indirectly to antagonize a protein phosphatase, thus favoring the inactive and more stable state of GLI1ΔN (Stecca and Ruiz i Altaba, 2009). p53 was also shown to inhibit GLI1 levels and function by inducing the transcriptional activation of PCAF in response to DNA damage (Mazzà et al., 2013). The intrinsic E3-ligase activity of PCAF leads to poly-ubiquitination in the C-terminus of GLI1 and subsequent degradation by the proteasome, thus attenuating the mitogenic and pro-survival properties of GLI1 in medulloblastoma (Mazzà et al., 2013). In another study, p53 was shown to sequester the TATA box binding protein (TBP)-associated factor 9 (TAF9), which is a component of the PCAF histone acetylase complex, and to prevent its association with GLI1 and GLI2 proteins (Yoon et al., 2015). Indeed, TAF9 was shown to physically interact with GLI1 at the residue L1052 in the transactivation domain in rhabdomyosarcoma and osteosarcoma cells. This interaction has been reported to enhance GLI-mediated transactivation of a specific pattern of target genes and, thus, transformation (Yoon et al., 2015).

The adaptor protein Numb is an evolutionary conserved protein implicated in cell fate specification, endocytosis, migration and stem cell maintenance (Gulino et al., 2010). Accumulating evidence suggests a role of Numb as a tumor suppressor in a variety of cancers. Loss of Numb has been described in medulloblastoma, where its downregulation has been associated with the hyperactivation of HH signaling that occurs as a consequence of GLI1 accumulation, leading to *in vitro* transformation and enhanced tumor growth and metastasis. Indeed, Numb acts as an adaptor protein that binds the E3 ubiquitin ligase Itch and releases it from its self-inhibitory conformation. Then, the catalytically active Itch interacts with a Serine residue at position 1060 (S1060) in a proline-rich motif in the C-terminal tail of GLI1, and recruits it into a complex with Numb. This results in the ubiquitination and proteasome-dependent degradation of GLI1 (Di Marcotullio et al., 2006, 2011). Consistent with these findings, HH-dependent medulloblastomas show an inverse correlation with levels of the Numb isoform p66, which is involved in neural differentiation and whose downregulation results in enhanced medulloblastoma CSC features (Abballe et al., 2018).

The tumor suppressor SNF5, a chromatin remodeling protein representing a core subunit of the ATP-dependent SWI-SNF complex, has been also shown to restrain the activity of HH signaling. Affinity purification–mass spectrometry performed in a HH-responsive mouse testicular epithelial cell line led to the identification of SNF5 among the top interactors of GLI1 (Jagani et al., 2010). This physical interaction has been reported to repress the activity of HH signaling via the control of chromatin structure at GLI1 target promoters. Notably, loss of SNF5, observed in malignant rhabdoid tumors, has been associated with derepression of transcriptional activity at *GLI1* locus and hyperactivation of HH signaling, which contributes to cancer formation (Jagani et al., 2010).

Small non-coding micro RNAs (miRNAs) are responsible for post-transcriptional regulation of gene expression via binding to 3′-UTRs of target mRNAs, resulting in their degradation or inhibition of translation. miRNAs are involved in several biological processes, such as proliferation, differentiation, development, and metabolism, and have been implicated in tumorigenesis by targeting developmental pathways involved in tumor formation (Ling et al., 2013; Oliveto et al., 2017). With regard to HH signaling, miRNAs appear to modulate the expression of GLI independently of PTCH/SMO. A previous study by Ferretti et al. (2008) showed that the tumor suppressor miR-324-5p acts as a negative regulator of HH signaling in cerebellar granule cell precursors (CGCP) by targeting and functionally suppressing *GLI1* mRNA. Chen et al. (2017) reported that derepression of miR-361 expression in prostate carcinoma cells directly inhibits GLI1 expression through the binding to the 3′-UTR of *GLI1* itself, thus restraining the malignant growth and invasiveness of prostate cancer. Downregulation of miR-361-3p has been also observed in retinoblastoma tissues. When overexpressed, miR-361-3p strongly restrains cell proliferation and CSC self-renewal of retinoblastoma cells, by repressing GLI1 and GLI3 (Zhao and Cui, 2019). miR-326 is another negative modulator of HH signaling, and it acts by inhibiting GLI2. Using a genome-wide expression profiling in embryonic lung explant cultures, Jiang and co-workers reported that miR-326 is part of a negative feedback loop in regulating the activity of HH signaling during lung development (Jiang et al., 2014). Indeed, while HH signaling induces the expression of miR-326 and of its host gene β-arrestin (Arrb1), miR-326 suppresses the expression of GLI2 (Jiang et al., 2014). Downregulation of miR-326 has been also recently identified as a critical feature of CSCs isolated from a mouse model of Shh-dependent medulloblastoma. Indeed, re-expression of miR-326 and Arrb1, which acts as a scaffold protein that facilitates the recruitment of p300 to target histones with consequent transcription activation, have been shown to induce a more differentiated phenotype by inhibiting HH-GLI signaling at multiple levels. First, miR-326 directly represses GLI2 by binding to its 3′-UTR, thus impairing the expression of several HH target genes; second, Arrb1 facilitates p300-mediated acetylation of GLI1 at K518, further inhibiting HH signaling (Miele et al., 2017).

### Epigenetic Modulators

Beside genetic modifications and other oncogenic inputs, alterations of the epigenetic machinery appear critical for tumor development. Recently, several lines of evidence pointed to an epigenetic control of HH signaling that occurs downstream of SMO and SUFU.

#### Bromodomain Proteins

Members of the bromo- and extra-terminal domain (BET) family of chromatin adaptors bind acetyl-lisine residues on open chromatin and facilitate gene transcription at super-enhancers through their interaction with the positive transcription elongation factor b (P-TEFb) and RNA polymerase II (PolII) (Mujtaba et al., 2007). Recently, the bromodomain-containing protein 4 (BRD4) has been reported to regulate HH signaling by directly binding to *GLI1* and *GLI2* promoters (Tang et al., 2014). Of note, its depletion has been associated with reduced survival of medulloblastoma CSC *in vitro* and tumor growth of a Ptch^+/-^ derived medulloblastoma allograft *in vivo* (Long et al., 2014). BET proteins have been also associated to GLI-dependent pancreatic cancer growth and stromal remodeling. Indeed, the antitumor effects of their inhibition occur most likely through cell-extrinsic mechanisms in the stromal compartment that affect GLI1 expression and alter the secretome of CAFs, leading to the suppression of PDAC growth and tumor-sphere formation in a paracrine manner (Yamamoto et al., 2016). However, BET proteins also physically associate with GLI proteins in PDAC cells in order to allow their occupancy on target gene promoters, denoting the existence of different mechanisms of regulation of the HH-GLI signaling pathway in PDAC tumors (Huang et al., 2016).

#### Chromatin Remodelers

Acetylation represents a crucial transcriptional event in various developmental and differentiation processes, being one of the modifications that impact chromatin remodeling and transcription, and its deregulation is involved in the development of several tumors. Histone acetyltransferases (HATs) and histone deacetylases (HDACs) catalyze, respectively, the addition or the removal of an acetyl group on lysine residues within the N-terminal histone tails as part of gene regulation (Bannister and Kouzarides, 2011). Several lines of evidence highlight the importance of HDACs in controlling the activity of HH signaling. A previous study showed that acetylation of Gli1 and Gli2 proteins inhibits their DNA binding ability, and that class I HDACs enhance the transcriptional activity of Gli1 through deacetylation of a lysine residue at position 518 (K518) (Canettieri et al., 2010). In physiological settings, the function of Gli1 is maintained through an integrated mechanism involving HDAC1 and REN_KCTD11_, which is an adaptor subunit of the Cullin-3 (Cul3)-based ubiquitin ligase complex, that targets HDAC1 for ubiquitination and subsequent proteasome-degradation, in order to ensure proper development and prevent tumorigenesis (Canettieri et al., 2010). Subversion of this mechanism due to HDAC overexpression or loss of REN, has been shown to induce persistent deacetylation of Gli1, leading to CGCP transformation and subsequent medulloblastoma growth (Canettieri et al., 2010). In support of this, selective inhibition of HDAC1/2 results effective in inhibiting HH signaling and reducing tumor growth in Shh-dependent medulloblastoma mouse models through increased acetylation of Gli1 at Lys518 (Coni et al., 2017). Recently, Gruber and co-workers showed that inhibition of class I HDACs represses the HH-GLI signaling in *Ptch1*-deficient mouse medulloblastoma cells not only by decreasing the DNA binding capacity of Gli1, but also contributing to efficient Gli3 repressor formation, thus implying for the first time a direct role of acetylation in Gli processing (Gruber et al., 2018). Indeed, previous reports linked the repressor activity of GLI3 to the Ski-dependent recruitment of HDAC (Dai et al., 2002), although whether and how it occurs was not addressed in this study.

In addition to HDAC1/2, the class II protein HDAC6 is found upregulated in medulloblastoma tumors, and its inhibition shows tumor suppressive effects in preclinical models of Shh-medulloblastoma. Remarkably, HDAC6 effects seem to be independent of GLI acetylation, and to occur most likely through the transcriptional control of GLI2 expression and stabilization of GLI3 protein (Dhanyamraju et al., 2015). In line with these findings, a recent study in multiple myeloma revealed that hyper-acetylation of GLI1 through pharmacological inhibition of class I and II HDACs leads to reduced tumor survival by decreasing nuclear accumulation and transcriptional activity of GLI1, and accelerating its proteasomal degradation (Geng et al., 2018).

Histone acetyltransferases have been also implicated in the regulation of non-canonical HH signaling. First, it has been shown that the HAT co-activator p300 functions as a crucial transcriptional checkpoint during morphogen-dependent development (Coni et al., 2013). Indeed, p300 has been shown to acetylate GLI2 at the conserved Lysine residue 757 (K757), preventing GLI2 recruitment to chromatin and thus inhibiting HH target gene expression (Coni et al., 2013). Second, PCAF plays opposing roles in modulating GLI1 activity, depending on microenvironmental conditions (Malatesta et al., 2013). Indeed, PCAF acts as a transcriptional cofactor of GLI1 in permissive conditions by increasing H3K9 acetylation at GLI target gene promoters, leading to brain tumor cell growth (Malatesta et al., 2013). Conversely, in non-permissive situations (i.e., genotoxic stress) PCAF switches to apoptotic activity and restrains HH functions and oncogenic properties by directly binding to GLI1 and promoting its ubiquitination and proteolysis via the E3 ligase activity (Mazzà et al., 2013).

### Protein Arginine Methyltransferases (PRMT)

Members of the protein arginine methyltransferase (PRMT) family regulate several cellular processes, including gene transcription, DNA repair, mRNA splicing and signal transduction. Aberrant activity of PRMT has been reported in several tumors (Yang and Bedford, 2013). PRMT1 can promote the transcriptional activity of GLI1 by inducing its methylation at residue Arg597, thus increasing binding of GLI1 to the promoter of its targets. Abrogation of GLI1 methylation reduces its oncogenic functions in PDAC (Wang et al., 2016). Studies in SHH-expressing gastric cancer cells and small cell lung cancer showed that GLI1 is methylated at three Arginine residues (515, 990 and 1018) by the methylosome protein 50 (MEP50)/PRMT5 complex. Methylations at Arg990 and Arg1018 act to inhibit the interaction of GLI1 with the E3 ligase complex ITCH/NUMB, leading to GLI1 stabilization, nuclear accumulation and transcriptional activation (Abe et al., 2019). The authors show also a positive correlation between the expression of MEP50/PRMT5 and that of GLI1 target genes in several HH-dependent cancers, and demonstrate that targeting of MEP50/PRMT5 complex may synergize with SMO inhibitors in suppressing cancer cell proliferation and invasion (Abe et al., 2019). By contrast, PRMT5 has been reported also to negatively affect GLI1 activation in endocrine organs (Gurung et al., 2013). Indeed, the tumor suppressor Menin was found to bind to *GLI1* promoter and recruits PRMT5 to repress GLI1 expression, at least partially through the PRMT5-catalyzed histone arginine methylation on histone H4 (H4R3m2). Thus, mutations in the *MEN1* gene result in activation of GLI1 through increased binding of transcriptionally active GLI1 at its promoter, leading to the development of neuroendocrine tumors (Gurung et al., 2013) (Figure 3).

## Implications for Cancer Therapy

To date, most of the efforts to inhibit HH-GLI signaling have been directed on the development of SMO inhibitors (SMOi), such as vismodegib (GDC-0449), sonidegib (NPV-LDE-225), saridegib (IPI-926), BMS-833923, glasdegib (PF-04449913) and taladegib (LY2940680) (Pietrobono and Stecca, 2018). These SMOi show improved potency, pharmacokinetics and tolerability compared to the natural steroidal alkaloid SMO antagonist cyclopamine, enhancing their clinical utility. The US Food and Drug Administration (FDA) and European Medicines Agency (EMA) have approved both vismodegib and sonidegib for treatment of locally advanced or metastatic BCC (LoRusso et al., 2011; Sekulic et al., 2012; Dummer et al., 2016). Furthermore, some of these SMOi have been successfully used for treating medulloblastoma, BCC and other advanced solid tumors (Rodon et al., 2014; Wagner et al., 2015; Pietanza et al., 2016; Stathis et al., 2017) as well as hematological malignancies (Martinelli et al., 2015; Savona et al., 2018).

However, acquisition of resistance due to specific missense mutations at SMO level (i.e., SMO-D437H) represents the major challenge to the success of therapies, as well documented in BCC (Atwood et al., 2015; Pricl et al., 2015; Sharpe et al., 2015). This limitation led to the development of novel SMOi with activity toward the mutated variants of SMO, such as MRT-92 (Hoch et al., 2015; Pietrobono et al., 2018). Besides, clinical trials with SMO antagonists in most solid tumors have failed most likely because in these tumors SMO is not the main oncogenic driver, and alternative oncogenic events could be responsible for SMO-independent GLI activation (i.e., RAS-ERK, PI3K-AKT-mTOR-S6K1 signaling, p53 loss, epigenetic alterations, etc.). In such cases, direct targeting of GLI might represent the best choice to improve the antitumor activity of these drugs.

In the last few years, several GLI inhibitors have been developed and tested. Some of them have been shown to directly interfere with DNA binding ability of GLI, including GANT58 and 61 (Lauth et al., 2007) and Glabrescione B (Infante et al., 2015), whereas others, such as HPI1-4 (Hyman et al., 2009) and ATO (Kim et al., 2010; Beauchamp et al., 2011), have been reported to modulate GLI processing and activation, trafficking and/or ciliary accumulation. However, with the exception of ATO, which is not specific for GLI and showed *in vitro* cytotoxicity (Yedjou and Tchounwou, 2007), none of these GLI inhibitors are good candidates for clinical studies.

Targeting alternative pathways responsible for non-canonical GLI activation together with SMO inhibition could therefore represent an intriguing strategy to allow the complete eradication of GLI-dependent tumors. In this section we provide examples of preclinical or clinical studies focused on combinatorial therapies with HH antagonists and inhibitors of the above-described oncogenic signaling pathways.

The RAS-RAF-MEK pathway is among the compensatory mechanisms that sustain HH-GLI signaling beyond SMO. Several preclinical studies demonstrated synergic effects between SMO inhibitors and MEK inhibitors in GLI-dependent tumors. For instance, co-administration of the SMO inhibitor SANT-1 with the MEK1 inhibitor PD325901 in prostate cancer cells characterized by hyperactivation of MAPK signaling, has been shown to reduce prostate cancer cell growth more than either single agent alone (Gioeli et al., 2011). Likewise, dual blockade of HH (with the SMOi cyclopamine) and MAPK signaling (with the MEKi U0126) showed increased efficacy in reducing proliferation and survival of cholangiocarcinoma cells (Jinawath et al., 2007). Furthermore, the existence of non-canonical activation of HH signaling by the MEK1/RSK2 axis responsible for GLI2 stabilization in multiple myeloma strongly supports the development of novel therapeutic strategies targeting both pathways. Indeed, simultaneous inhibition of GLI with GANT58 and RSK2 with SL0101 has been reported to synergistically reduce GLI2 levels, enhancing apoptosis of multiple myeloma cells (Liu et al., 2014). Hyperactivation of the MAPK signaling pathway has been also recently related to the acquisition of resistance to vismodegib in medulloblastoma by promoting a niche of SMOi-insensitive cells in which the RAS-RAF-MEK pathway sustains tumor proliferation, while HH pathway becomes dispensable (Zhao et al., 2015). In line with this, previous work by Ji and co-workers showed that pharmacological inhibition of MEK1/2 with UO126 reduced GLI activation in PDAC cells resistant to SMOi, thus representing an efficient strategy to contrast the non-canonical activation of HH signaling (Ji et al., 2007).

Given that activation of various RTKs, including EGFR, converges on the RAS-RAF-MEK-ERK pathway, is not surprising that their inhibition in combination with that of HH signaling could overcome or delay resistance mechanisms observed after prolonged treatment with SMOi. EGFR signaling has been shown to cooperate with HH signaling in various contexts (Palma and Ruiz i Altaba, 2004; Palma et al., 2005; Schnidar et al., 2009; Eberl et al., 2012) and to induce GLI activation through the MAPK pathway (Kasper et al., 2006). In support of this, several preclinical studies in the last decade have been focused on combining SMOi with RTK inhibitors, such as those of EGFR, in order to improve the antitumor response. For instance, combination treatment with the SMOi saridegib and the EGFR inhibitor cetuximab abrogates tumor growth and delays tumor recurrence in mouse xenograft models derived from metastatic head and neck squamous cell carcinoma (Bowles et al., 2016). Likewise, co-administration of GANT61 with erlotinib (an EGFR inhibitor) has been reported to impair tumor initiating properties of pancreatic cancer cells, and to reduce tumor growth in HH-driven BCC mouse models (Eberl et al., 2012). Treatment with SMOi has been also shown to enhance the efficacy of EGFR inhibitors in non-small cell lung cancer (Ahmad et al., 2013), prostate cancer (Mimeault et al., 2006, 2007) and glioblastoma CSCs (Eimer et al., 2012).

PI3K-AKT-mTOR signaling represents a promising therapeutic strategy for GLI-dependent tumors, given its involvement in enhancing transcriptional activity and increasing nuclear localization of GLI. Sharma et al. (2015) demonstrated the efficacy of combining the SMO inhibitor sonidegib with the dual PI3K/mTOR inhibitor NVP-BEZ-235 on pancreatic CSC survival and tumorigenicity. Similarly, combined inhibition of GLI with GANT61 and mTOR with rapamycin has been reported to improve the effects of single agent alone on pancreatic cancer cell viability and CSC self-renewal, and to suppress *in vivo* growth of pancreatic cancer xenografts (Miyazaki et al., 2016). In another study, Wang et al. (2012) showed that inhibition of HH pathway with vismodegib in esophageal adenocarcinoma cell lines and mouse xenografts has a more potent inhibitory effect in presence of the mTOR inhibitor everolimus (RAD001), implying that PI3K/mTOR could be responsible for the acquisition of resistance of esophageal adenocarcinoma to SMOi. Consistently, treatment with the PI3K inhibitor GDC-0941 strongly inhibited the growth of vismodegib-insensitive tumor models (Dijkgraaf et al., 2011). By applying gene expression profiling of sonidegib-resistant versus sonidegib-sensitive medulloblastomas, Buonamici and collaborators identified a number of PI3K target genes that were enriched only in resistant samples, suggesting that hyperactivation of this pathway could contribute to the acquisition of resistance. To support this, authors provided evidence that co-administration of the PI3K inhibitor NVP-BKM120 and sonidegib significantly delays the onset of resistance and tumor regrowth, without inhibiting growth of already established resistant tumors (Buonamici et al., 2010). Combination of sonidegib and NVP-BKM120 has been also shown to abrogate glioblastoma tumor growth by inducing mitotic catastrophe and apoptosis (Gruber-Filbin et al., 2013). Thus, the use of dual HH and PI3K-AKT-mTOR inhibitors (Yang et al., 2015) could represent a promising therapeutic approach to counteract the emergence of resistance in those tumors.

Given the involvement of TGF-β pathway in the activation of GLI proteins independent of PTCH/SMO, combined treatment modalities with inhibitors of SMO and TGF-β could be beneficial for GLI-dependent tumors. For instance, Dennler et al. (2007) demonstrated that pharmacologic blockade of TGF-β signaling in SMOi-resistant PDAC cells expressing high levels of GLI, reduces GLI2 expression with a subsequent repression of cancer cell proliferation. Furthermore, a recent study reported that combined inhibition of TGF-β with SD208 and of GLI with GANT61 could reverse chemoresistance in colorectal cancer, and prevent CSC relapse in patients after chemotherapy (Tang et al., 2018).

Combined inhibition of HH signaling and HH-related protein kinases could also represent a suitable therapeutic approach for treating GLI-dependent tumors, especially those in which non-canonical activation of GLI limits the efficacy of SMOi (Montagnani and Stecca, 2019). For instance, pharmacological inhibition of DYRK1B has been reported to repress GLI expression downstream of SMO and subsequent *in vivo* tumor growth of both SMOi-sensitive and resistant pancreatic cancer cells (Gruber et al., 2016).

Bromodomain-containing protein 4 is another attractive therapeutic target for GLI-dependent tumors, since it has been directly involved in the transcriptional regulation of GLI. Of note, administration of the BET inhibitors JQ1 or I-BET-151 suppresses the HH pathway transcriptional output in several HH-driven tumors, such as BCC, medulloblastoma and atypical teratoid rhabdoid tumors, even when the emergence of primary or acquired resistance mechanisms compromises the efficacy of SMO inhibitors (Long et al., 2014; Tang et al., 2014).

Targeting HDAC represents an additional intriguing therapeutic option, as members of HDAC have been reported to activate HH signaling by deacetylating GLI proteins. Indeed, the use of the selective HDAC1/2 inhibitor mocetinostat inhibits HH signaling and reduces tumor growth in preclinical models of SHH-dependent medulloblastoma (Coni et al., 2017). Further, the FDA-approved HDAC inhibitor vorinostat has been proposed in combination with small molecule inhibitors of aPKC for treating advanced BCC, since aPKC appears to mediate the recruitment of HDAC1 to GLI1 (Mirza et al., 2017). HDAC inhibitors have been also successfully used in combination with SMOi to improve the therapeutic outcome of several GLI-dependent tumors. For instance, co-administration of the HDAC inhibitor SAHA with vismodegib has been shown to suppress tumor growth in multiple aerodigestive cancer cell lines (Chun et al., 2015), and the dual HDAC/HH pathway inhibitor NL-103 effectively overcomes vismodegib resistance conferred by SMO-M2 and SMO-D473H mutants by downregulating the expression of GLI2 (Zhao et al., 2014). Of note, the small molecule HDAC inhibitor 4SC-202 displayed high efficacy in both vismodegib-sensitive and SUFU-depleted SMOi-resistant medulloblastoma cells, indicating that inhibition of HDAC activity could bypass the acquired resistance toward SMOi (Gruber et al., 2018).

## Conclusion

A wealth of data indicate that the GLI transcription factors are regulated by several oncogenic signaling pathways and inputs in addition or independent of canonical PTCH-SMO signaling. These findings in part explain the failure of clinical trials with SMO antagonists, urging the development of novel therapeutic strategies to inhibit non-canonical HH signaling.

In general, canonical activation of HH-GLI signaling occurs in cancers with mutations in *PTCH1* and *SMO*, such as BCC and SHH-type medulloblastoma, most of which are sensitive to SMO antagonists. As summarized in this review, several mechanisms of non-canonical activation of HH signaling in cancer have been reported (Table 1). In the majority of cancer types, including glioblastoma, melanoma, colon, prostate, esophageal and pancreatic cancers, one or more non-canonical HH signaling modes can take place. Thus, it is critical to understand specific, non-canonical mechanisms of HH pathway activation in a given tumor type before starting anti-cancer treatment. At this purpose, the development of *in vitro* and *in vivo* preclinical models, such as patient-derived cell lines, organoids and xenografts (PDX), will be needed to test the role of each aberrantly activated signaling pathway and provide personalized therapeutic options for individual patients. In addition, it will be equally important to establish sensitive and validated biomarkers of HH-GLI pathway activation, such as reliable antibodies against human GLI1 and GLI2 and their phosphorylated forms. For treatment involving SMO inhibitors (alone or in combination with other pathway inhibitors), a better biomarker for SMO activation should be developed, such as phospho-specific antibody to monitor SMO phosphorylation (Chen et al., 2011). The availability of such tools and biomarkers will allow to screen cancer patients and select those showing SMO and GLI activation, increasing the subset of cancer patients who will likely respond to HH-GLI pathway inhibition and to monitor the efficacy of therapy.

In summary, both canonical HH signaling and the confluence of multiple SMO-independent oncogenic pathways can regulate the activity of the GLI transcription factors. Thus, a more effective therapeutic approach to fight cancers harboring non-canonical HH pathway activation would be to inhibit the GLI rather than SMO. Often in a given cancer type, canonical and non-canonical HH pathway activation co-exist. In some cancer types, more than one mechanism of non-canonical HH activation occurs simultaneously. Therefore, combined targeted therapy will be more effective than single treatment in blocking GLI-dependent tumor growth and progression.

## Author Contributions

SP and BS conceptualized the content, and reviewed and edited the manuscript. All authors wrote and approved the manuscript.

## Conflict of Interest Statement

The authors declare that the research was conducted in the absence of any commercial or financial relationships that could be construed as a potential conflict of interest.
